# Differential Expression of Proteoglycans in Tissue Remodeling and Lymphangiogenesis after Experimental Renal Transplantation in Rats

**DOI:** 10.1371/journal.pone.0009095

**Published:** 2010-02-05

**Authors:** Heleen Rienstra, Kirankumar Katta, Johanna W. A. M. Celie, Harry van Goor, Gerjan Navis, Jacob van den Born, Jan-Luuk Hillebrands

**Affiliations:** 1 Immunology Section, Department of Cell Biology, University Medical Center Groningen, University of Groningen, Groningen, The Netherlands; 2 Nephrology Division, Department of Internal Medicine, University Medical Center Groningen, University of Groningen, Groningen, The Netherlands; 3 Department of Pathology, Academic Medical Center, University of Amsterdam, Amsterdam, The Netherlands; 4 Pathology Division, Department of Pathology & Medical Biology, University Medical Center Groningen, University of Groningen, Groningen, The Netherlands; Centre National de la Recherche Scientifique, France

## Abstract

**Background:**

Chronic transplant dysfunction explains the majority of late renal allograft loss and is accompanied by extensive tissue remodeling leading to transplant vasculopathy, glomerulosclerosis and interstitial fibrosis. Matrix proteoglycans mediate cell-cell and cell-matrix interactions and play key roles in tissue remodeling. The aim of this study was to characterize differential heparan sulfate proteoglycan and chondroitin sulfate proteoglycan expression in transplant vasculopathy, glomerulosclerosis and interstitial fibrosis in renal allografts with chronic transplant dysfunction.

**Methods:**

Renal allografts were transplanted in the Dark Agouti-to-Wistar Furth rat strain combination. Dark Agouti-to-Dark Agouti isografts and non-transplanted Dark Agouti kidneys served as controls. Allograft and isograft recipients were sacrificed 66 and 81 days (mean) after transplantation, respectively. Heparan sulfate proteoglycan (collXVIII, perlecan and agrin) and chondroitin sulfate proteoglycan (versican) expression, as well as CD31 and LYVE-1 (vascular and lymphatic endothelium, respectively) expression were (semi-) quantitatively analyzed using immunofluorescence.

**Findings:**

Arteries with transplant vasculopathy and sclerotic glomeruli in allografts displayed pronounced neo-expression of collXVIII and perlecan. In contrast, in interstitial fibrosis expression of the chondroitin sulfate proteoglycan versican dominated. In the cortical tubular basement membranes in both iso- and allografts, induction of collXVIII was detected. Allografts presented extensive lymphangiogenesis (p<0.01 compared to isografts and non-transplanted controls), which was associated with induced perlecan expression underneath the lymphatic endothelium (p<0.05 and p<0.01 compared to isografts and non-transplanted controls, respectively). Both the magnitude of lymphangiogenesis and perlecan expression correlated with severity of interstitial fibrosis and impaired graft function.

**Interpretation:**

Our results reveal that changes in the extent of expression and the type of proteoglycans being expressed are tightly associated with tissue remodeling after renal transplantation. Therefore, proteoglycans might be potential targets for clinical intervention in renal chronic transplant dysfunction.

## Introduction

Chronic transplant dysfunction (CTD) explains the majority of long-term loss of transplanted kidneys [1], [2]. Although considerable improvement has been made in overall graft survival due to improved prevention and treatment of acute rejection, the rate of long-term renal graft loss has remained unchanged over more than a decade. CTD is the overall outcome of tissue remodeling processes in multiple intrarenal structures (*i.e.* the intrarenal arteries, the glomeruli and the interstitium leading to transplant vasculopathy (TV), focal glomerulosclerosis (FGS) and interstitial fibrosis (IF), respectively [2]–[4] and is the resultant of various underlying causes [5]. TV is presumed to result from activation of the vascular endothelium, leading to the activation and migration of vascular smooth muscle cells (SMCs) and the development of an occlusive neointima in the lumen of the arteries [6]. The neointima consists of smooth muscle cells, extracellular matrix and inflammatory cells [7]. FGS presumedly results from defects in the filtration barrier, which is formed by podocytes, the glomerular basement membrane (BM) and endothelial cells [8]. IF results from the accumulation of extracellular matrix synthesized by infiltrating and proliferating interstitial myofibroblasts. To date, due to the lack of knowledge on the pathogenesis of tissue remodeling leading to CTD, no effective therapies are available to prevent or treat CTD. The identification of molecules involved in pathological tissue remodeling after renal transplantation might provide novel targets for intervention.

Proteoglycans are glycoconjugates that play an important role in tissue remodeling. They consist of a core protein with one or more carbohydrate side chains (*i.e.* glycosaminoglycans, GAGs) attached. Depending on the composition of these GAGs, different types of proteoglycans can be distinguished: heparan sulfate (HS), chondroitin sulfate (CS), dermatan sulfate (DS), and keratan sulfate proteoglycans. The extracellular matrix HS proteoglycans collagen type XVIII (collXVIII), perlecan and agrin are components of BMs of various tissues [9]–[11]. The CS/DS proteoglycan versican is a major extracellular matrix component which is not expressed in BMs. Depending on the sulfation patterns of the carbohydrate side chains, HS and CS/DS proteoglycans are capable of binding and presenting a variety of proteins like chemokines and growth factors and are involved in various cell-cell and cell-matrix interactions [12].

Using a rat renal transplant model for chronic transplant dysfunction [13]–[15], we sought to determine the spatial expression of HS (collXVIII, perlecan, agrin) and CS/DS (versican) proteoglycans and its association with lymphangiogenesis. We used specific (semi-)-quantitative immunofluorescent (double)labeling to identify the spatial expression of proteoglycans and lymphatics in non-transplanted kidney, isografts and allografts. We established a clear spatial relationship between the presence of HS and CS/DS proteoglycans in allografts and the development of CTD. Allografts were characterized by marked tubulointerstitial lymphangiogenesis coinciding with enhanced perlecan expression. Both the magnitude of lymphangiogenesis and perlecan expression correlated with IF development and impaired graft function.

## Methods

### Rats

Inbred female (175–210 gram) and male (200–225 gram) Dark Agouti (DA) rats were obtained from Harlan (Horst, the Netherlands) and inbred male Wistar Furth (WF) rats (240–295 gram) from Charles River Laboratories Inc. (l'Arbresle, Cedex, France).

### Ethics Statement

All animals received care in compliance with the Principles of Laboratory Animal Care (NIH Publication No. 86-23, revised 1985), the University of Groningen guidelines for animal husbandry (University of Groningen, the Netherlands), and the Dutch Law on Experimental Animal Care.

### Kidney Transplantation and Experimental Groups

Female DA kidneys were orthotopically transplanted into male recipients as described previously [14]. Cold ischemic time ranged from 16 to 38 min. Warm ischemic time ranged from 19 to 32 min. Recipients received cyclosporine A (5 mg/kg bodyweight) (Sandimmune, Novartis, the Netherlands) subcutaneously on the first 10 days after transplantation. The contralateral native kidney was removed 8 to 14 days after transplantation. Total follow-up was 12 wks or shorter in case animals had to be sacrificed due to graft failure. The following experimental groups were included: control (non-transplanted) DA kidneys (n = 5), DA-to-DA isografts (n = 5), and DA-to-WF allografts (n = 11). Clinical variables of recipients within these groups have been described in detail elsewhere [14], [15]. Briefly, isograft recipients were sacrificed at day 81 [70–84] (mean [range]) and allograft recipients at day 66 [40–84] after transplantation. The allograft recipients showed significantly increased plasma creatinine levels measured at time of sacrifice compared with isograft recipients (119 [93–139] µmol/L *vs.* 42 [34]–[50] µmol/L, respectively, p<0.005, Mann-Whitney test). In addition, allograft recipients developed severe proteinuria compared with isograft recipients (110 [9–262] *vs.* 18 [9]–[43] mg/day, respectively, p<0.05, Mann-Whitney test).

### Immunofluorescence

Four-micron frozen sections fixed in acetone or 4% formaldehyde were blocked for endogenous peroxidase activity with 0.03% H_2_O_2_ if appropriate. For some stainings the sections were blocked with normal goat, rabbit or mouse serum. Sections were incubated for 1 hr with the following primary antibodies: rabbit anti-mouse collagen XVIII (NC11,kindly provided by Dr. T. Sasaki, Shrines Hospital for Children, Portland, OR, USA), mouse anti-rat perlecan (10B2, kindly provided by Dr. Couchman, Biomedicine Institute, University of Copenhagen, Denmark), sheep anti-rat agrin (Gr14) [16], mouse anti-heparan sulfate stub region (F69-3G10, Tokyo, Japan), mouse anti-HS (JM-403) [17], [18], mouse anti-rat CD31 (TLD-3A12, BD Pharmingen, the Netherlands), and rabbit anti-LYVE-1 (Millipore, USA). Binding of primary antibodies was detected by incubating the sections for 30 min with secondary antibodies diluted in PBS with normal rat serum: rabbit anti-mouse HRP (DAKO, Belgium), goat anti-rabbit FITC (SouthernBiotech, USA), and rabbit anti-sheep HRP (DAKO). HRP activity was visualized using the TSA™ Tetramethylrhodamine System (PerkinElmer LAS Inc., USA). Nuclei were stained with DAPI.

### L-Selectin Binding Assay with Enzymatic Pre-Treatments

L-selectin-IgM chimeric protein, consisting of the extracellular domain of human L-selectin linked to an IgM Fc-tail [19] was allowed to bind for 1 hr. L-selectin binding was detected by incubation with rabbit anti-human IgM HRP (DAKO) for 30 min, followed by the use of the TSA™ Tetramethylrhodamine System. Heparitinase I (EC4.2.2.8), and chondroitinase ABC (EC4.2.2.4) (both from Seikagaku, Tokyo, Japan) were used to digest the side chains of the heparan sulfate and chondroitin sulfate proteoglycans, respectively. To this end, prior to some L-selectin binding assays, enzymatic pretreatments were performed with heparitinase I (0.05 U/ml) and/or chondroitinase ABC (1 U/ml) in acetate buffer (50 mM C_2_H_3_O_2_Na, 5 mM CaCl_2_·2H_2_O, 5 mM MgCl_2_·6H_2_O, [pH 7.0 for heparitinase I, pH 8.0 for chondroitinase ABC]) for 1 hr at 37°C.

### Fluorescence Microscopy

Fluorescence microscopy was performed using a Leica DMLB microscope (Leica Microsystems, Rijswijk, the Netherlands) equipped with a Leica DC300F camera and LeicaQWin 2.8 software. Confocal imaging was performed with a Leica TCS SP2 confocal laser scanning microscope equipped with the Leica Confocal Software package (version 2.61).

### Quantification of Protein Expression in Stained Tissue Sections

Semiquantitative scoring of proteoglycan expression was performed independently by two observers (HR and KK) and the mean value of both observers was used. In the rare cases discrepancies were identified between the values of both observers, the respective sections were re-evaluated in the presence of a third observer (JvdB) until consensus was reached. Both intensity of the staining as well as the stained surface area of the structures in the specific renal compartments (*i.e.* intima, media, neointima, Bowman's capsule, glomerular BM, mesangial matrix, interstitial matrix and tubular BMs) were scored separately in a semi-quantitative manner on a scale ranging from 0–4. For staining intensity the following grading was used (relative to the strongest staining observed in the specific renal compartment of interest): 0 = no staining, 1 = weak staining, 2 = moderate staining, 3 = strong staining, 4 = most intense staining observed. For surface area stained the following grading system was used (relative to the total area of the specific renal compartment of interest): 0 = no staining, 1 = 0–25% area positive, 2 = 26–50% area positive, 3 = 51–75% area positive, 4 = 76–100% area positive. Quantification of perlecan, CD31 and LYVE-1 in the outer medulla was performed in four overview photomicrographs of randomly selected fields (magnification 320×). The total area stained was quantified using ImageJ 1.41 (Rasband, W.S., ImageJ, U.S. National Institutes of Health, Bethesda, Maryland, USA) which was downloaded from http://rsb.info.nih.gov/ij/download.html).

### Statistics

Differences between non-transplanted control kidneys, isografts and allografts in total area stained for perlecan, CD31 and LYVE-1 were tested with a Mann-Whitney test. Spearman correlation analysis was performed to correlate the magnitude of lymphangiogenesis to interstitial fibrosis and renal function parameters. p<0.05 was considered statistically significant. Statistics were performed using GraphPad Prism 5.00 for Windows (GraphPad Software Inc., USA).

## Results

### Chronic Tissue Remodeling in Renal Grafts

**Figure 1:**
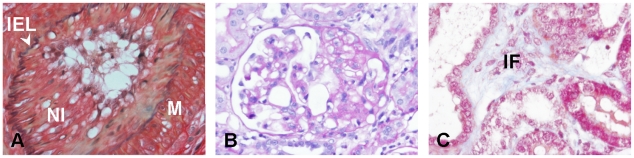
Allografts present severe development of transplant vasculopathy (A), focal glomerulosclerosis (B) and interstitial fibrosis (C). (A) Intrarenal artery with a neointima (Verhoeff staining [elastic laminae: black; collagen: red; smooth muscle cells: yellow]). (B) Glomerulus with a sclerotic lesion (periodic acid-Schiff staining [glycans in connective tissue: purple-magenta]). (C) Part of the tubulointerstitium with a fibrotic area (Masson's trichrome staining [collagen: blue]). Stainings were performed on 2 µm formalin-fixed paraffin sections. Abbreviations: IEL: internal elastic lamina; IF: interstitial fibrosis; M: media; NI: neointima. Magnification 400×.

The rat renal allografts showed severe CTD with TV, FGS and IF (Figure 1), as reported previously [14], [15]. The isografts developed some interstitial remodeling characterized by tubular atrophy and IF, but to a much lesser extent than observed in the allografts [14], [15]. Development of TV and FGS was minimal in isografts. The scant tissue remodeling observed in isografts was mainly related to the transplantation procedure rather than to the short-term use of cyclosporine as in the contralateral native kidney (removed at nephrectomy after cyclosporine treatment) no tissue remodeling was detected (not shown).

### Heparan Sulfate Proteoglycan Expression in Non-Transplanted Control Kidneys

**Figure 2:**
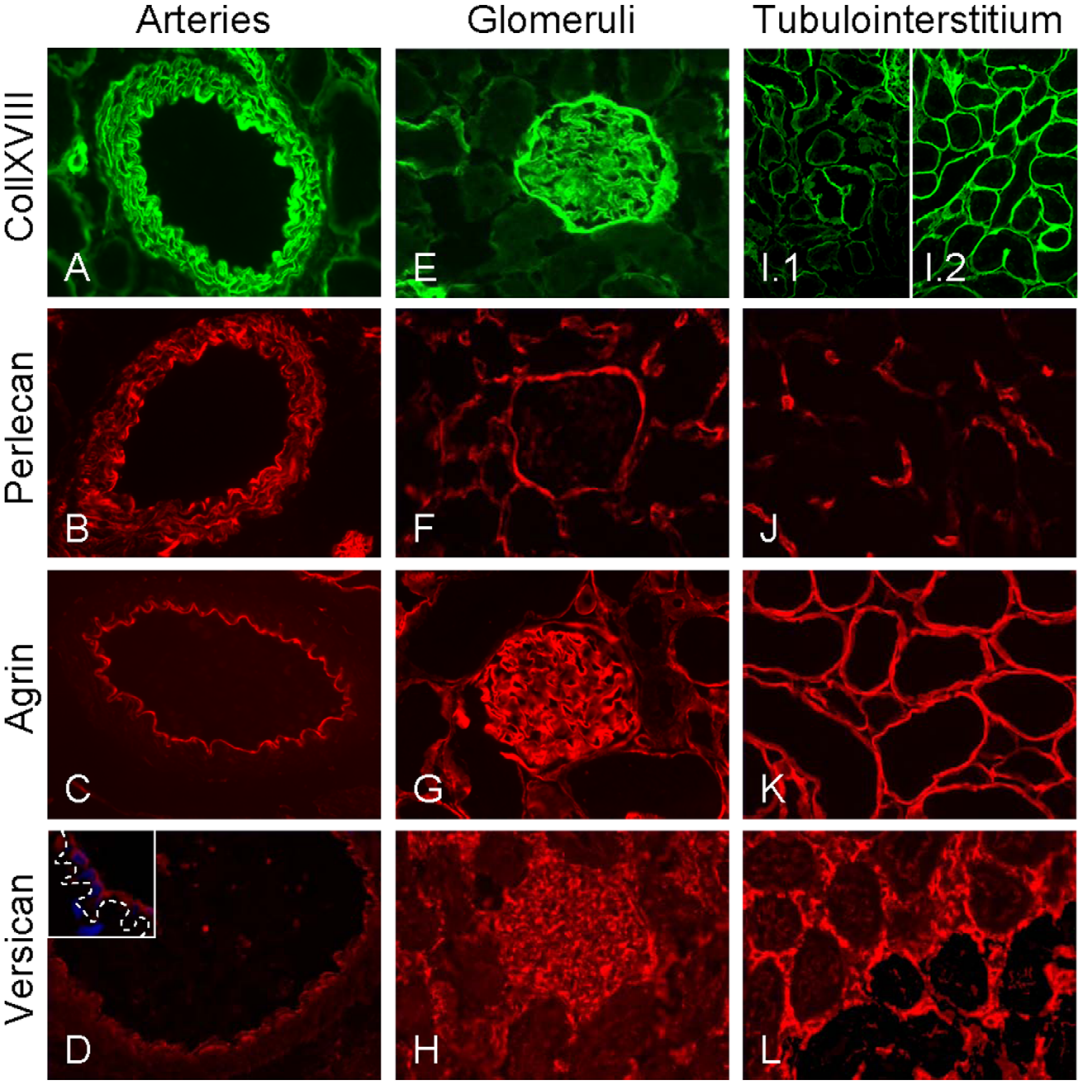
Proteoglycan expression in arteries, glomeruli and tubulointerstitium of non-transplanted control kidneys. All proteoglycans were strongly expressed in the intima of arteries (A–D). HS proteoglycans were located in the subendothelial BM while versican was located in the endothelial cell membrane (insert D, confocal image, magnification 3780×). The BMs of vascular SMCs in the media showed a strong expression of collXVIII and a patchy expression of perlecan (A and B). In the glomeruli (E–H), the glomerular BM moderately expressed collXVIII (E) while perlecan was virtually absent (F) whereas agrin was abundantly present (G). All HS proteoglycans were expressed in Bowman's capsule but only minimally in the mesangial matrix (E–G). Dotted staining pattern for versican suggested expression by podocytes. (H). In the tubulointerstitium (I–L), tubular BMs minimally expressed collXVIII and perlecan in the cortex (I.1 and J). Compared with the cortex, collXVIII expression was increased in medullary tubular BMs (I.2). Perlecan was moderately to strongly expressed in peritubular capillaries (J). Agrin was uniformly expressed in tubular BMs (K). Versican was not present in tubular BMs but strongly expressed in the tubulointerstitial matrix (L). Magnifications: A–G, H & J: 640×; I: 320×.

In non-transplanted DA control kidneys, the HS proteoglycans collXVIII, perlecan, and agrin, as well as the CS/DS proteoglycan versican were strongly expressed in the intima of the arteries (Figure 2A–D). More specifically, the HS proteoglycans were located in the subendothelial BM whereas versican was located in the apical endothelial cell membrane (Figure 2D, inset). The BM of vascular SMCs in the media were characterized by a strong expression of collXVIII and a patchy expression of perlecan (Figure 2A and B). The arterial expression of proteoglycans was similar in arteries in isografts and arteries without TV in allografts (not shown).

In the glomerular BM of non-transplanted control kidneys, expression of collXVIII was moderate whereas perlecan was virtually absent (Figure 2E and F). In contrast to collXVIII and perlecan, agrin was abundantly present (Figure 2G). All three HS proteoglycans were strongly expressed in the Bowman's capsule but only minimally in the mesangial matrix (Figure 2E–G). A patchy glomerular versican expression pattern was observed suggesting non-glomerular BM staining (Figure 2H). Double labeling for versican and the mesangial cell marker CD90 (Thy-1) [20] did not reveal co-localization indicating non-mesangial versican origin (not shown). Double labeling for versican and CD31 revealed minor co-localization of versican and glomerular ECs (not shown). Since the limited endothelial versican expression could not account for the majority of glomerular versican expression, these data suggest podocyte origin of glomerular versican. These observations are in line with previous data showing that glomerular versican is expressed by both podocytes and glomerular endothelium [21], [22].

In the tubular BM, expression of collXVIII was only minimal in the cortical regions (Figure 2I.1) but more pronounced in the medullary regions (Figure 2I.2). Agrin was strongly expressed in all tubular BMs in a uniform fashion (Figure 2K). The tubular BMs were virtually devoid of perlecan expression but we observed moderate to strong expression of perlecan in the peritubular capillaries (Figure 2J) as well as in larger vessels (Figure 2B). Although versican was not present in the tubular BM, it was strongly expressed in the interstitial matrix (Figure 2L), where all three HS proteoglycans were absent.

Table 1 summarizes the semi-quantitative scoring of proteoglycan expression (both intensity and surface area) in the intrarenal arteries, glomeruli and interstitium. Below we describe the significant changes in expression of HS proteoglycans and versican in renal isografts, and allografts with CTD.

**Table 1:** Proteoglycan expression in the specific renal compartments in non-transplanted control kidneys, isografts, and allografts.

	Artery			Glomerulus			Tubulointerstitium		
	Intima	Media	NI	BC	GBM	MM	Matrix	TBM	
								cortex	med
**CollXVIII**									
control	3 (3)	2 (4)		3 (4)	2 (4)	1 (4)	0	½ (4)	1½ (4)
isograft	3 (3)	2 (4)		3 (4)	2 (4)	1 (4)	½ (1)	***2 (4)***	2 (4)
allograft	3 (3)	2 (4)	3 (4)	3 (4)	1–3 (4)	***1–3 (4)***	½ (1)	***2 (4)***	2 (4)
**Perlecan**									
control	3 (2)	1–3 (4)		1–3 (3)	½ (1)	½ (1)	0	½ (1)	½ (1)
isograft	3 (2)	1–3 (4)		1–3 (3)	***2–3 (2)***	***1–3 (2)***	1 (1)	1 (2)	1 (2)
allograft	3 (2)	1–3 (4)	3 (4)	1–3 (3)	***3 (4)***	***1*** *–* ***3 (4)***	1 (1)	1 (2)	1 (2)
**Agrin**									
control	3 (4)	½ (1)		1–3 (3)	3 (4)	1 (2)	0	2½ (4)	3 (4)
isograft	3 (4)	½ (1)		1–2 (3)	3 (4)	1 (2)	0	2–3 (4)	2–3 (4)
allograft	3 (4)	1–2 (1)	1–3 (2)	1–3 (3)	3½ (4)	1 (2)	0	2–3 (4)	2–3 (4)
**Versican**									
control	2 (4)	1 (3)		0	0	0	***3 (4)***	0	0
isograft	2 (4)	1–2 (3)		0	0	0	***2–3 (4)***	0	0
allograft	2 (4)	1–2 (3)	2 (1)	0	0	0	***2***–***3 (4)***	0	0

Semi-quantitative scores (ranging from 0–4) of proteoglycan expression presented as the staining intensity and, between parentheses, the surface area stained (as described in detail in the *Methods* section). Scores given are the group means of the different grafts analyzed. Numbers of grafts analyzed are: non-transplanted control, n = 5; isografts, n = 5; allografts, n = 11. The values represented in ***bold/italic*** indicate differentially expressed proteoglycans in the various groups as discussed in more detail in the *Results* section. Abbreviations: NI, neointima; BC, Bowman's capsule; GBM, glomerular basement membrane; MM, mesangial matrix; TBM, tubular basement membrane; med, medulla.

### Differential Proteoglycan Expression in Isografts Compared with Control Kidneys

The expression profile of proteoglycans in renal isografts was mostly similar to the expression profile in control kidneys except for changes in the glomerular and tubular BMs (Table 1). In the glomerular BM, perlecan expression was increased compared with control kidneys, but to a far lesser extent than observed in allografts (described below). In the tubular BM of isografts, an increased cortical expression of collXVIII and slight induction of perlecan was observed. The expression of agrin in the tubular BM remained strong alike control kidneys, although after transplantation the expression was slightly interrupted. In regions with IF (only limited present compared with allografts), an increased expression of versican was observed.

### Differential Proteoglycan Expression in Neointima and FGS versus IF in Allografts

**Figure 3:**
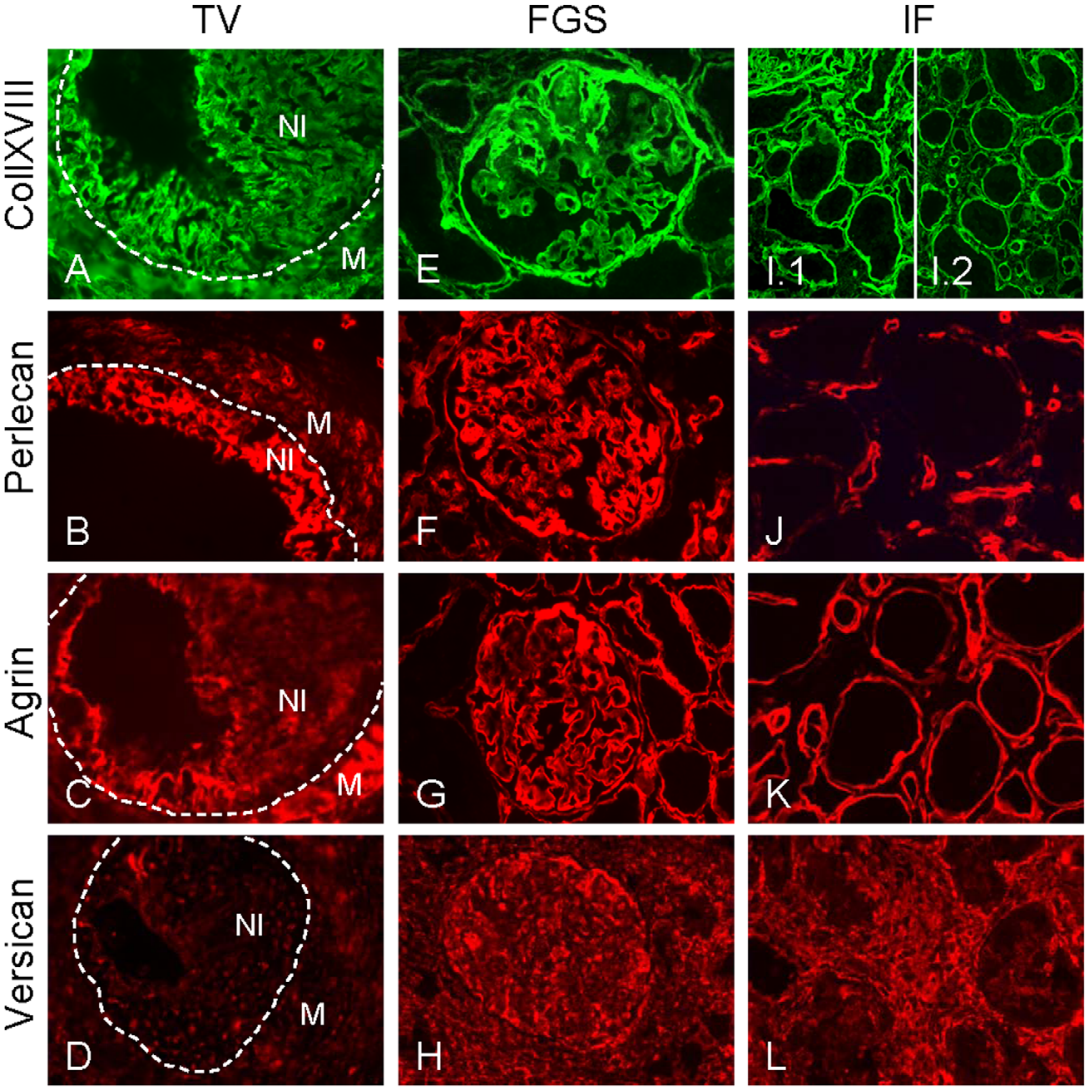
Proteoglycan expression in transplant vasculopathy (TV), focal glomerulosclerosis (FGS) and tubulointerstitial fibrosis (IF) in allografts. In the neointima in TV, collXVIII and perlecan were strongly expressed (A and B). Expression of agrin and versican was less prominent in the neointima but their expression was slightly upregulated in the media (compared with non-transplanted control tissue) (C and D). Dotted lines indicate the internal elastic lamina. In the glomeruli (E–H), expression of collXVIII in the glomerular BM was variable with strong expression in glomerulosclerotic lesions (E). Perlecan was strongly induced in the glomerular BM (F). Agrin expression remained similar to its expression in glomerular BMs in non-transplanted control tissue (G). Versican staining was comparable with non-transplanted control tissue (H). In the tubulointerstitium (I–L), collXVIII (I) and perlecan (J) were minimally present in IF in which agrin expression was absent (K). CollXVIII was clearly expressed by tubular BMs in cortical (I.1) and medullary (I.2) regions at similar levels. Versican was strongly expressed in IF (L). In the cortical tubular BM, collXVIII was strongly expressed with a strong, but slightly interrupted, expression of agrin (I and K). Perlecan was only weakly expressed in the tubular BM but strongly expressed in peritubular capillaries (J). Magnification 640×.

In arteries with TV in allografts, strong expression of collXVIII and perlecan was observed in the newly-formed neointima (Figure 3A and B). The expression of agrin and versican in the neointima was less prominent than collXVIII and perlecan, and varied in intensity within a single neointima. Agrin and versican expression in the media was slightly upregulated compared with non-transplanted control kidneys (Figure 3C and D).

In sclerotic lesions of FGS, we also observed a strong expression of collXVIII and perlecan (Figure 3E and F). Within a single glomerulus differential expression of collXVIII expression was detected and contained both areas with decreased or strongly increased expression compared with control kidneys and isografts (Figure 3E). Agrin was not differently expressed in these lesions (Figure 3G) compared with glomerular BM staining in control kidneys. Compared with control kidneys and isografts, no altered expression of versican was observed in the glomeruli of allografts (Figure 3H). In the glomerular BM, and probably also podocytes of allografts, an impressive induction of perlecan expression was detected compared with its expression in control kidneys and isografts (Figure 3F).

In contrast to the neointima and glomerulosclerotic lesions, collXVIII and perlecan were minimally expressed in IF with complete absence of agrin (Figure 3I–K). However, collXVIII/agrin and perlecan were clearly expressed in the tubular BM and peritubular capillaries, respectively (Figure 3I–K) as described in more detail below. In contrast to the HS proteoglycans, we observed a massive expression of versican in IF (Figure 3L). These data indicate that in neointimal and glomerular lesions HS proteoglycans dominate, whereas in regions with IF CS/DS proteoglycans are more prominent.

### Increased Expression of collXVIII in Cortical Tubular Basement Membranes in Allografts

The expression profile of proteoglycans in the cortical tubular BM was similar in allografts and isografts (Table 1). In the allografts, increased expression of collXVIII and a slight induction of perlecan was detected (Figure 3I and J). Increased collXVIII expression was observed in both cortical (Figure 3I.1) and medullary (Figure 3I.2) tubular BMs. Agrin remained strongly expressed; however, in a less homogeneous pattern compared with control kidneys (Figures 2K and 3K).

### Proteoglycan Core Proteins Expressed in Allografts Contain Functional Glycosaminoglycan (GAG) Side Chains

**Figure 4:**
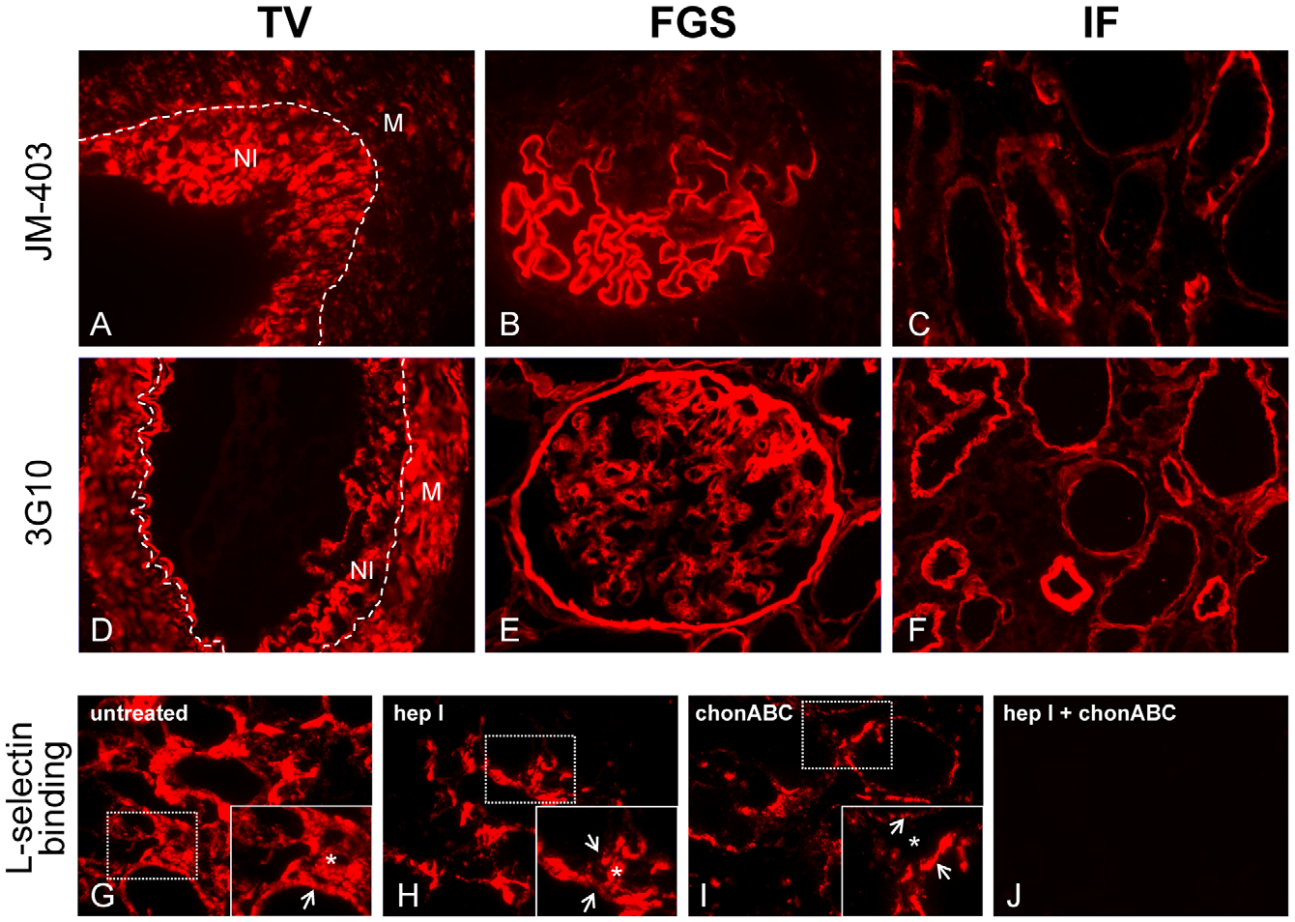
Proteoglycan core proteins expressed in transplant vasculopathy, glomerulosclerosis and interstitial fibrosis in allografts contain functional glycosaminoglycan side chains. Neointimal cells in TV (A), glomerular BMs in non-sclerotic areas (B) and tubular BMs in IF (C) express heparan sulfate domains with N-unsubstituted glucosamine residues as recognized by antibody JM-403. Following heparitinase treatment presence of heparan sulfate stub regions was identified in medial and neointimal cells in TV (D), in glomerular BMs (E) and in tubular BMs (F) using antibody F69-3G10. Dotted line in panel A and D represents the internal elastic lamina. Abbreviations: M: media; NI: neointima. L-selectin-IgM chimeric protein binding in the tubulointerstitium in no pre-treated sections (G), sections pre-treated with heparitinase I [hep I] (H), sections pre-treated with chondroitinase ABC [chonABC] (I) and sections pre-treated with both heparitinase I and chondroitinase ABC (J). Insets show high power magnifications of the framed areas. Arrows: tubular BMs, asterisks: interstitium.

In order to determine whether the proteoglycans expressed in allografts contain HS GAG side chains, stainings with the antibodies JM-403 and 3G10 were performed. JM-403 recognizes heparan sulfate domains with N-unsubstituted glucosamine residues [17], [18]. As shown in Figure 4A–C, neointimal cells in TV (A), glomerular BMs in non-sclerotic areas of glomeruli (B) and tubular BMs in IF (C) indeed expressed heparan sulfate domains with N-unsubstituted glucosamine residues, thereby confirming the presence of HS side chains. As JM-403 only recognizes a specific subset of HS side chains, not all HS glycosaminoglycans present will be detected using JM-403. Therefore, we additionally performed stainings using antibody F69-3G10. F69-3G10 recognizes HS stubs that remain attached to the proteoglycan core protein following heparitinase treatment. As shown in Figure 4D–F, F69-3G10 staining revealed presence of HS stubs in medial and neointimal cells in TV (D), in glomerular BMs (E) as well as tubular BMs (F), also confirming presence of HS glycosaminoglycans on the proteoglycan core proteins. Finally, to demonstrate that the glycosaminoglycan side chains are capable of binding L-selectin (as an example of a natural ligand), L-selectin binding assays were performed. As shown in Figure 4G, L-selectin binding in the tubulointerstitium was detected on the tubular BMs as well as in the interstitium. To determine whether L-selectin preferentially binds to HS or CS/DS proteoglycans, sections were pre-incubated with heparitinase I and/or chondroitinase ABC. Following heparitinase I pre-treatment, only interstitial L-selectin binding was preserved indicating preferential binding of L-selectin to HS proteoglycans in tubular BMs (Figure 4H). Following chondroitinase ABC pre-treatment, only L-selectin binding to tubular BMs was preserved indicating preferential binding of L-selectin to CS/DS proteoglycans in the interstitium (Figure 4I). Sections pre-treated with both heparitinase I and chondrointinase ABC were completely devoid of L-selectin binding (Figure 4J).

### Abundant Lymphangiogenesis Is Related to Increased Expression of Perlecan

**Figure 5:**
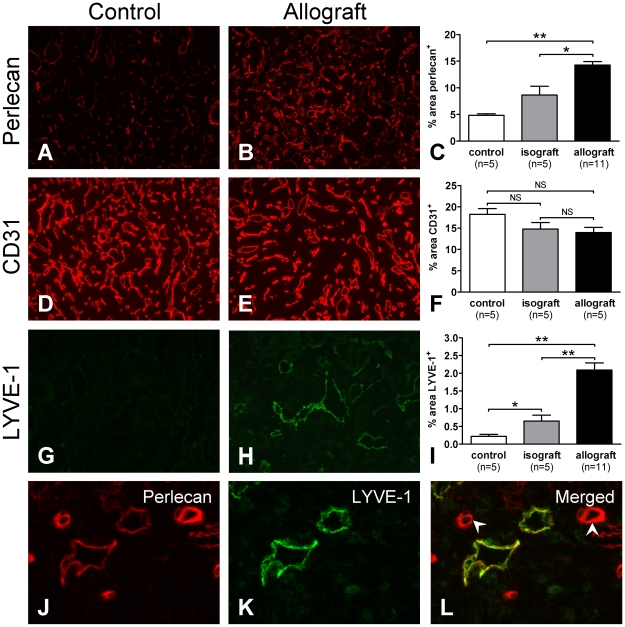
Lymphangiogenesis in the tubulointerstitium is associated with perlecan expression. Expression of perlecan in allografts was significantly increased compared with non-transplanted control kidneys and isografts (A–C). After transplantation (of both iso- and allografts), the area with CD31 expression slightly decreased (D–F) (NS: not significant). In allografts, LYVE-1 expression was significantly increased compared with non-transplanted control kidneys and isografts (G–I). Double staining for perlecan and LYVE-1 revealed that perlecan is expressed in association with lymphatic endothelium in the newly-formed lymphatics (J–L). Arrowheads indicate peritubular capillaries strongly positive for perlecan but negative for LYVE-1. C, F and I represent the quantification of surface area stained for perlecan, CD31 and LYVE-1, respectively. Magnification A, B, D, E, G, H: 320×; J–L: 640×. *p<0.05, **p<0.01

In the interstitium of allografts we observed a significant increase in expression of perlecan in a capillary-like pattern (Figure 5A–C). Since perlecan has been related to angiogenesis [23]–[26], we analyzed whether the increased perlecan expression was associated with the formation of new peritubular capillaries. However, quantification of interstitial CD31 staining revealed that allografts do not contain an increased number of peritubular capillaries (Figure 5D–F). Instead, we observed a non-significant decrease in area stained positively for CD31 per given surface area in both allografts and isografts, which might be associated with enlargement of the renal graft after transplantation due to removal of the contralateral kidney [15]. When staining for the lymphatic marker LYVE-1 [27], we observed a marked increase in the number of lymph vessels in the allografts (Figure 4G–I) indicative of lymphangiogenesis. In isografts, we also observed an increase in LYVE-1 staining but to a significant lesser extent than observed in allografts (Figure 5I). Double labeling for perlecan and LYVE-1 revealed that the newly-formed lymph vessels all express perlecan in their BM (Figure 5J–L). In addition, allografts also displayed abundant presence of capillary-like structures that were strongly positive for perlecan but LYVE-1 negative, indicating an overall upregulation of perlecan in pre-existing peritubular capillaries as well (Figure 5J–L). The observed increase in perlecan expression in isografts and allografts positively correlated with the severity of IF (Spearman r = 0.5580, p = 0.0475) as well as serum creatinine levels (Spearman r = 0.7770, p = 0.0004 [8 wk] and Spearman r = 0.8182, p = 0.0011 [12 wk]) and proteinuria (Spearman r = 0.6714, p = 0.0061 [8 wk] and Spearman r = 0.7510, p = 0.0031 [12 wk]).

### Lymphangiogenesis in Renal Grafts Correlate with IF and Impaired Graft Function

**Figure 6:**
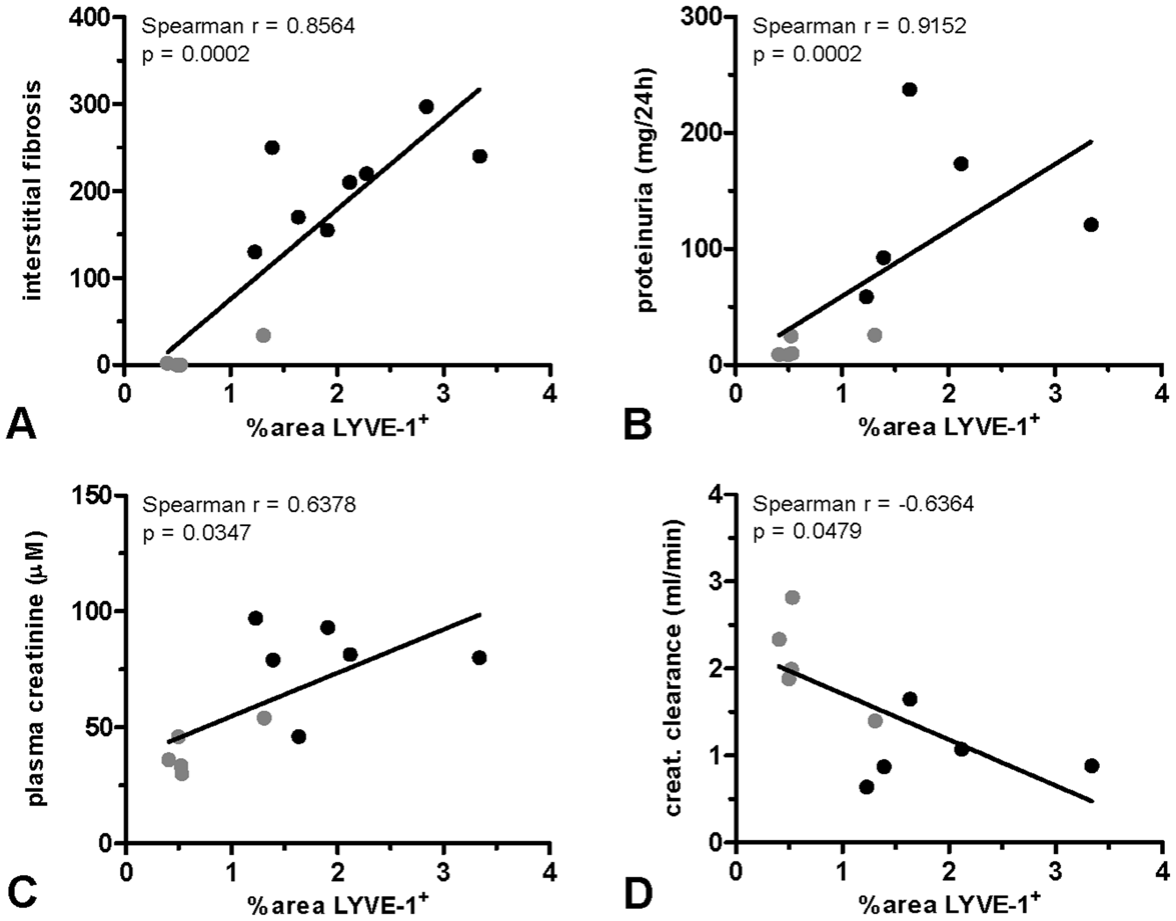
Lymphangiogenesis in the tubulointerstitium correlates with severity of interstitial fibrosis (A), proteinuria (B), plasma creatinine levels (C) and creatinine clearance (D). Interstitial fibrosis, proteinuria (8 wks post transplantation), plasma creatinine levels (8 wks post transplantation) and creatinine clearance (8 wks post transplantation) were determined as recently described [14], [15]. LYVE-1 expression was quantified as described in the *Methods* section. (gray circle isografts, black circle allografts).

In order to analyze whether the magnitude of tubulointerstitial lymphangiogenesis correlates with the severity of IF and graft function, Spearman correlation analyses were performed in which data from both the isografts and allografts were included. As shown in Figure 6A, the magnitude of lymphangiogenesis showed a significant positive correlation with the severity of IF. When analyzing proteinuria and plasma creatinine levels (measured at 8 wks after transplantation), also these functional variables turned out to be positively correlated with the magnitude of lymphangiogenesis at sacrifice (Figures 6B & C, respectively). Similarly, plasma creatinine levels at sacrifice positively correlated with lymphangiogenesis (Spearman r = 0.8571, p = 0.0137). Increased plasma creatinine levels translated into reduced creatinine clearance, which was inversely correlated with the magnitude of lymphangiogenesis (Figure 6D).

## Discussion

This study is the first to demonstrate the extensive involvement of proteoglycans in tissue remodeling in experimental CTD, demonstrating marked changes in proteoglycan expression in the intrarenal arteries, glomeruli and interstitium. The following key observations were made. First, whereas HS proteoglycans dominate in neointimal lesions in TV and in FGS, the CS/DS proteoglycan versican dominates in IF. Second, glomerular remodeling is associated with an impressive induction of perlecan expression in the glomerular BM. Third, the HS proteoglycan content becomes increased in cortical tubular BMs, especially due to increased collXVIII expression. Finally, allografts are characterized by marked tubulointerstitial lymphangiogenesis which correlates with IF development and impaired graft function.

Some earlier work demonstrated increased HS polysaccharides in fibrotic and sclerotic lesions in vessels, interstitium and mesangium of chronic renal transplant dysfunction [28], along with increased GAG-mediated chemokine binding [29], [30]. However, proteoglycan core-proteins were not identified in those studies. In non-transplant renal diseases, tubular upregulation of collXVIII/endostatin was reported in a number of experimental models [31]–[33]. Mesangial expression of perlecan and agrin was reported in human diffuse mesangial sclerosis [34], in diabetic nephropathy [35], [36], and some other human glomerulopathies with mesangial expansion [37]. Concerning proteoglycan expression in the neointima, both perlecan and versican have been shown to be present in neointimal lesions formed after experimental or human stenting or denudation or related to atherosclerosis [38]–[40]. The neointima in arteries of human cardiac allografts contain versican [41]. The striking similarities in proteoglycan expression in transplantation-unrelated kidney diseases and chronic renal allograft dysfunction suggest comparable matrix remodeling programs. This might indicate that anti-fibrotic treatments in various kidney diseases might also reduce chronic transplant dysfunction.

We showed differential expression of HS proteoglycans in neointimal lesions and FGS on one hand, and CS/DS proteoglycans in IF on the other, suggesting the existence of spatial (*i.e.* compartment-specific) proteoglycan responses during the development of CTD with potentially variable biological effects. Expression of the CS/DS proteoglycan versican in IF is likely involved in leukocyte recruitment and infiltration by its L-selectin-binding capacity [30], [42]. The abundant versican expression in IF supports our previous finding that L-selectin in the interstitium binds to CS/DS side chains and not HS side chains [43]. Moreover, the high L-selectin-binding capacity of CS/DS proteoglycans in IF fits well with our observation that most leukocyte infiltration was observed in interstitial regions and to a far lesser extent in neointimal lesions and within the glomeruli. The marked expression of versican in IF is probably produced by interstitial myofibroblasts [44], [45]. Tubulointerstitial versican might contribute to the activation and proliferation of intra- and extrarenal myofibroblasts and may also mediate their recruitment. In line with this, we recently demonstrated that ∼53% of interstitial myofibroblasts in IF are derived from extrarenal sources [14] and may originate from a population of recirculating fibrocytes. Fibrocytes are mesenchymal progenitor cells exhibiting morphological characteristics of hematopoietic stem cells, monocytes and fibroblasts and have the capacity to differentiate into α-SMA-expressing myofibroblasts which is promoted by TGF-ß [46]. Although HS proteoglycans have been shown to mediate hematopoietic progenitor cell homing [47] this needs to be experimentally proven for CS/DS proteoglycans.

In contrast to interstitial myofibroblasts in IF, neointimal SMCs in experimental renal allografts are solely derived from an intrarenal source, probably the arterial media [14]. In the current study, we observed a strong expression of perlecan in the neointima. Perlecan expression in arteries has been associated with inhibition of SMC proliferation and reduced intimal hyperplasia [25], [39], [48]–[50] which favours for a role of perlecan in neointima stabilization. However, data reported by others indicate that arterial HS proteoglycans can actually activate SMC proliferation by modulating the function of basic fibroblast growth factor (bFGF/FGF2) [51]. Although clear expression of collXVIII was observed in the neointima, its potential role in neointima formation is as yet unknown.

After transplantation, we observed a strong induction of perlecan in the glomerular and peritubular capillary BMs. Peritubular capillaries play an essential role in graft rejection [52]. Upon capillary inflammation, endothelial cells become activated and changes occur in the BM, like splitting and multi-layering [53], [54]. The response in peritubular capillaries is similar to that observed in glomerular capillaries, and the thickened BM might be the resultant of processes associated with endothelial cell death and regeneration [53], [54]. Capillary BM changes are related to our previous data indicating endothelial chimerism *(i.e.* presence of recipient-derived endothelial cells) in glomerular and peritubular capillaries in CTD [14]. Both endothelial chimerism and perlecan expression in capillaries could be essential in capillary endothelial regeneration [23]. Perlecan in capillary BM might thus play a role in maintaining the capillary endothelial integrity but also contribute to the inflammatory response [30].

We observed a major increase of collXVIII expression in the tubular BM after renal transplantation in both iso- and allografts. The integrity of the tubular BM and its changes are involved in inflammation, phenotypic changes of tubular epithelial cells, and the development of IF and tubular atrophy [29], [55]–[57]. Tubular epithelial cells can contribute to IF via epithelial-to-mesenchymal transition (EMT) in which epithelial cells transdifferentiate into interstitial myofibroblasts [58]–[60]. CollXVIII and (weakly expressed) perlecan in the tubular BM could play a role in the EMT process by binding of chemokines and growth factors resulting in a concentration gradient in the tubular BM [61]. This gradient might then direct migration of tubular epithelial cells into the interstitium during EMT. In line with this, preliminary data indeed suggest increased binding capacity of HS proteoglycans for FGF-2 in the tubular BM in allografts (not shown).

The more interrupted and less uniform expression of agrin in tubular BMs after transplantation supports the assumption that agrin normally plays a role in anchoring tubular epithelial cells, and focal loss of agrin could therefore be related to migration of transdifferentiated tubular cells in EMT or tubular atrophy [62], [63]. In addition to tubular atrophy and EMT, proteoglycan expression in the tubular BM could be involved in binding of L-selectin, thereby facilitating inflammatory responses in tubules [30], [64]. The potential causal role of BM HS proteoglycans in tubular atrophy or EMT are under current investigation in HS proteoglycan mutant mice.

We showed a marked induction of lymphangiogenesis in allografts, which was accompanied by the expression of perlecan at the abluminal side of lymphatic endothelium. Recovery of renal lymph drainage is shown to occur as early as 24 hours after renal transplantation [65], suggesting that lymph drainage and the process of lymphangiogenesis after renal transplantation is of potential functional relevance. However, it is still a matter of debate whether lymphangiogenesis and potential development of lymphoid structures in renal grafts is beneficial or detrimental to clinical outcome. Lymph vessels could be beneficial by mediating the drainage of extravasated fluid and the export of leukocytes [66]–[68]. On the other hand, lymph vessels and additional development of lymphoid structures could also perpetuate the inflammatory response [66], [67], [69]–[71]. We observed a clear correlation between the magnitude of lymphangiogenesis and severity of IF, suggesting that new lymph vessel formation may enhance the fibrotic process by stimulating the inflammatory process. This is supported by recent findings in diabetic nephropathy indicating that lymphangiogenesis is associated with inflammatory cell infiltration and progression of IF [72]. In our study, increased lymphangiogenesis correlated with reduced graft function suggesting that therapies that target *de novo* lymphatic formation might contribute to improved graft function. The existence of a causal relation between lymphangiogenesis and loss of graft function, however, needs to be established in future studies. The expression of perlecan in close proximity of lymphatic endothelial cells suggests a functional role for perlecan in lymphangiogenesis. This is supported by results from studies performed in a mouse model for regenerating skin which suggest that perlecan is involved in lymphatic endothelial cell migration, lymphatic organization and maturation [73]. In addition, also versican, which was abundantly present in the interstitium, might play a role in lymphangiogenesis [74].

In conclusion, we identified increased spatial expression of HS and CS/DS proteoglycans in the intrarenal arteries, glomeruli and tubulointerstitium undergoing extensive tissue remodeling associated with CTD in renal allografts. Compartment-specific expression of proteoglycans in CTD might translate into compartment-specific responses to therapy. In line with this concept, we recently reported a differential response in renal allograft remodeling to aldosterone receptor blockade using spironolactone in which spironolactone ameliorated TV and FGS but not IF [15]. The potential role of proteoglycans in the spironolactone-induced effects are currently under investigation.

Although our results are descriptive in nature, the observed differential expression of proteoglycans in renal allografts most likely also have functional consequences as the proteoglycan core proteins were shown to have GAGs that were able to bind L-selectin. Preliminary data furthermore suggest altered endogenous expression of natural proteoglycan ligands (such as FGF-2, HB-EGF, and L-selectin on leukocytes). As a resultant, the bioavailability of these ligands, which orchestrate tissue remodeling and inflammation, is most likely modulated due to altered proteoglycan expression as well as GAG side chain modifications.

Based on our results we propose that proteoglycans could be targets for intervention to ameliorate CTD. As an example, antibodies recognizing, and thereby blocking, specific HS-motifs/domains may inhibit leukocyte extravasation resulting in reduced inflammation. Also generated small inactive chemokine fragments might be used to block the HS proteoglycan-binding sites of their *in vivo* active counterparts thereby making the HS proteoglycans less bioactive. Alternatively, we suggest the possibility to produce small HS-mimetics which may target more specifically a particular component of HS/heparin bioactivity [75]. Therefore, focus should now be on the identification of the precise functional role of proteoglycans in chronic tissue remodeling after renal transplantation followed by exploration of the feasibility to use proteoglycans as targets for therapeutic intervention to ameliorate the development of CTD.
